# Replicability of the Curvature Effect as a Function of Presentation Time and Response Measure in Japanese Observers

**DOI:** 10.1177/2041669520915204

**Published:** 2020-03-26

**Authors:** Tomoki Maezawa, Tomoyuki Tanda, Jun I. Kawahara

**Affiliations:** Department of Psychology, Hokkaido University

**Keywords:** curvature effect, object preference, presentation time, rating scale

## Abstract

Although objects with curved contours are generally preferred over those with sharp-angled contours, the strength of this preference varies according to several factors. In the present study, non-Western Japanese observers viewed and rated their preferences (e.g., *liking* or *attractiveness*) for real and meaningless objects with curved or sharp-angled contours. We varied the presentation time (90 ms vs. until a response was received) and the response measure (like/dislike vs. 1–100 rating scale). When using like/dislike ratings, a preference for curved objects was found only when images of real objects were presented briefly (90 ms), whereas this effect was reversed (i.e., increased preference for sharp-angled contours) when using the 1 to 100 scale under the until-response condition. In addition, the curvature effect was not observed for real objects when the like/dislike rating and the until-response condition were employed or when the 1 to 100 scale and 90 ms presentation time were used. The curvature effect for meaningless objects remained unstable regardless of presentation time or response measure. Similar to the preference for real objects, a preference for sharp-angled objects was observed when preference was measured using a 1 to 100 rating scale. Taken together, the present findings indicate that the preferences for curved objects were situation-dependent in Japanese observers.

Of the various visual properties, researchers have identified several that could affect the perceived attractiveness of an object. For example, symmetric, brighter, typical, or perceptually fluent objects are preferred compared with asymmetric, darker, atypical, or perceptually nonfluent objects (Lakens et al., 2013; Perrett, 2012; Perrett et al., 1999; Winkielman et al., 2006). One of the basic properties that contribute to the apparent attractiveness of an object is curvature of contour. In fact, human preference toward an object shows a positive bias for objects with curved contours compared with objects with sharp-angled contours (Bar & Neta, 2006^2007^; Silvia & Barona, 2009; Vartanian et al., 2019). Although this bias is known and has been termed the curvature effect (Palumbo & Bertamini, 2016), the factors underlying this phenomenon remain unclear.

An original study by Bar and Neta (2006) demonstrated the curvature effect using a relatively small sample size (*n* = 14) and an image-rating task. In that study, the participants viewed a single image that was briefly presented (84 ms) from among a pool of 360 images that consisted of curved or sharp-angled real or abstract objects and were then asked to indicate whether they liked or disliked the object using a two-alternative forced-choice response (like/dislike). The participants had a significantly higher preference for curved objects than for sharp-angled objects, and, ultimately, the biased preference toward the curved objects represented the curvature effect. This phenomenon remains stable regardless of whether the objects are real or abstract. Bar and Neta (2006, 2007, 2008) further suggested that this effect is caused by a primitive sense of threat when viewing sharp-angled contours because it triggers a negative response. Although the primary focuses of subsequent studies have not been directly relevant to this hypothesis, they have investigated the curvature effect using a variety of experimental methodologies (Gómez-Puerto et al., 2016) in Western and non-Western populations (Gómez-Puerto et al., 2018; Velasco et al., 2016) and nonhuman animals, such as apes (Munar et al., 2015).

Although previous studies have considered this biased preference (e.g., in terms of liking, attractiveness, approachability, and comfort) toward curved contours to be a stable phenomenon (e.g., Bertamini et al., 2016; Palumbo & Bertamini, 2016; Vartanian et al., 2019), the robustness of the curvature effect needs to be further examined in humans because a preference for a curved object can be substantially influenced by various types of stimuli and testing circumstances (Leder et al., 2011; Munar et al., 2015). Importantly, a preference for an object is affected by whether it is a real or meaningless stimulus (Bar & Neta, 2006; Bertamini et al., 2016; Leder & Carbon, 2005) and whether it is accompanied by a positive or negative emotional valence (Leder et al., 2011; Vartanian et al., 2013). For example, this preference is reduced when objects without semantic content are presented and disappears when objects with negative emotional valence are presented (Bar & Neta, 2006, 2007; Leder et al., 2011). In general, the results from a series of relevant studies and their associated effect sizes are diverging (Table 1). Not surprisingly, one’s preference for an object is modulated by the visual properties associated with contour (Palumbo & Bertamini, 2016) as well as one’s familiarity with and the semantic content of objects (Bar & Neta, 2006), emotional valence (Leder et al., 2011; Vartanian et al., 2013), one’s experience viewing curved lines (Silvia & Barona, 2009; Vartanian et al., 2019), and artistic expertise or openness to experience (Cotter et al., 2017).

**Table 1.** Study Characteristics and Effect Sizes.

Reference	Culture	Stimuli (object)	Exposure duration	Response measure	Preference	Effect size (*t* test, ANOVA)
Bar & Neta (2006)	–	Real, Abstract	84 ms	Like/dislike	Curved	*d*s *>* 0.67
Bar & Neta (2007)	–	Real, Abstract	85 ms	Like/dislike	Curved	*d*s > 0.50
Silvia & Barona (2009)	Western	Abstract	2 s	1–9 scale	Curved	–
Leder et al. (2011)	Western	Real, Abstract	84 ms	Like/dislike	Curved	${\text{η}}_{\text{p}}^{\text{2}}$ = .23
		Real (positive valance)	84 ms	Like/dislike	Curved	–
		Real (negative valance)	84 ms	Like/dislike	n.s.	–
Vartanian et al. (2013)	Western	Real	3 s	Beautiful/not beautiful	Curved	–
Munar et al. (2015)	Western	Real	80 ms	Choose preferred one	Curved	*d* = 0.81
			Until-response	Choose preferred one	n.s.	*d* = 0.03
Bertamini et al. (2016)	–	Abstract	Until-response	0–100 scale	Curved	${\text{η}}_{\text{p}}^{\text{2}}$ = .44
Palumbo & Bertamini (2016)	Western	Abstract	120 ms	Like/dislike	Curved	${\text{η}}_{\text{p}}^{\text{2}}$ = .68
			Until-response	0–100 scale	Curved	${\text{η}}_{\text{p}}^{\text{2}}$ = .48
Cotter et al. (2017)	Western	Abstract	2 s	1–7 scale	Curved	–
Corradi et al. (2019b)	Western	Real	34 ms, until-response	Choose preferred one	n.s.	–
		Real	48, 84, 150, 300 ms	Choose preferred one	Curved	–
		Abstract	84, 150, until-response	Choose preferred one	Curved	–
Gómez-Puerto et al. (2018)	Western and African	Real	80 ms	Choose preferred one	Curved	*d*s *>* 0.86
Vartanian et al. (2019)	Western	Real	Until-response	Beautiful/not beautiful	Curved	–
Corradi et al. (2019a)	Western	Real, Abstract	84 ms	Choose preferred one	Curved	–
Experiment 1 (the present study)	Asian (Japan)	Real, Abstract	90 ms	Like/dislike	Curved (only real objects)	*d* = 0.30
Experiment 2			Until-response	Like/dislike	n.s.	${\text{η}}_{\text{G}}^{\text{2}}$ = .002*d* = 0.04
Experiment 3			90 ms	1–100 scale	Sharp-angled (only abstract objects)	*d* = 0.19
Experiment 4			Until-response	1–100 scale	Sharp-angled	*d*s > 0.17

*Note*. Effect sizes (e.g., Cohen’s *d*) reflect the difference between curved and sharp-angled preference not attributable to chance. The values were calculated if the studies reported standard deviation and *df* values. ANOVA = analysis of variance.

In addition, the duration of stimulus presentation time is thought to be a critical factor that modifies a preference for curved objects (Corradi et al., 2019b; Munar et al., 2015). The idea that the potential threats associated with sharp-angled contours contribute to the curvature bias (Bar & Neta, 2006, 2007, 2008) is based on the assumption that the rapid extraction of information from an object at a low processing level underlies preferences (Bar, 2003; Bar et al., 2006). Accordingly, a brief exposure to a stimulus, which would prevent top-down processing, has been commonly applied in experiments investigating the curvature effect (e.g., Gómez-Puerto et al., 2018). Recently, Corradi et al. (2019b) examined the robustness of the curvature effect related to stimulus presentation times, including the use of an until-response exposure duration as an extreme condition. In that study, the participants’ preferences for real curved objects increased when the target was presented briefly (84 ms and 150 ms), whereas the effects were reversed when the exposure duration was prolonged such that the curvature effect was reduced. An analogous effect related to a long presentation time was observed in a relevant study performed by Munar et al. (2015). Presumably, a longer presentation time would allow the visual system to underestimate initially extracted information (VanRullen, 2007) by subsequently overriding top-down processing (Wyatte et al., 2012). Thus, Munar et al. (2015) hypothesized that the rating of objects based on semantic information would be more elaborate when the presentation time is extended (see Leder et al., 2011). Taken together, these findings suggest that the presentation time of a stimulus might modulate the curvature effect.

The aforementioned effects of presentation time on curvature preference (Corradi et al., 2018; Munar et al., 2015) apparently provide evidence that is not exactly similar to previous findings about the robustness of the curvature effect. However, the aforementioned studies employed different experimental methodologies in terms of response measures, compared with the original study by Bar and Neta (2006), which makes direct comparisons of the results extremely difficult. More specifically, Bar and Neta (2006) asked participants to indicate whether they liked or disliked a single presented image, whereas Corradi et al. (2019b) and Munar et al. (2015) asked participants to choose one of two simultaneously presented images. Corradi et al. (2019b) employed a brief presentation time (34 ms) and found that the participants did not have sufficient time to inspect the structure of the contour. Other authors have suggested that increasing the similarity of two stimuli exacerbates the difficulty of discriminating between them (e.g., Kramer & Ritchie, 2016; Nachmias, 2011). Because perceptual fluency directly affects preference judgments, it is reasonable to assume that a preference for an object would be modulated by the response measure used to record one’s preference. Thus, preference may differ when using a like/dislike forced-choice measure (e.g., Bar & Neta, 2006) versus a rating scale (e.g., Westerman et al., 2012), which convey implicit and explicit decisions for participants (e.g., Bertamini et al., 2016; Dewey & Knoblich, 2014), respectively.

Therefore, the present study was designed to examine the robustness of the curvature effect (Bar & Neta, 2006) by systematically manipulating stimulus presentation time and the response measures used to indicate a preference (like/dislike forced choice vs. a rating scale). The four experiments in the present study (Table 1) relied on comparisons between different preference tasks for assessing real and abstract images. Although Palumbo and Bertamini (2016) studied similar comparisons between a two-alternative forced-choice task and a rating scale task, these authors did not use real object images and also manipulated the visual complexity of concavities in the abstract objects using exposure durations of 120 ms.

Thus, the present study examined whether presentation time would affect the preferences for real and meaningless curved objects using two different types of response measures. Participants in Experiment 1 were required to make a like/dislike response after a single image of a real or meaningless object with a curved or sharp-angled contour was briefly presented (90 ms). Subsequently, using the same procedure as Experiment 1, Experiment 2 employed an until-response condition duration for the presentation time with a like/dislike response measure, Experiment 3 included a 90-ms exposure duration and a visual analog scale that ranged from 1 to 100 for the response measure, and Experiment 4 presented a single image of an object until a response was received in given amount of time, and the participants were required to respond using the visual analog scale ranging from 1 to 100. Moreover, the present study included participants from Japan who had never been included in a similar study (Table 1). Although several previous studies (e.g., Carbon, 2010) have attempted to demonstrate differences in the curvature effect across cultures, the influence of ethnicity remains unclear. Thus, the collection of data from divergent populations will be beneficial for a better understanding of a preference for certain types of curvature.

## Experiment 1

The purpose of Experiment 1 was to examine whether participants would prefer curved objects to sharp-angled objects following a brief presentation time when reporting preference (i.e., liking) using a like/dislike forced-choice response. Real objects and meaningless objects were used, and, thus, Experiment 1 also examined effects of the meaningfulness of objects on a preference for a curved contour. Each participant briefly viewed an image (90 ms) and then immediately rated their preference for it. Thus, the present study constituted a direct replication of the original study by Bar and Neta (2006) while recruiting participants from a different population.

### Method

#### Participants

Experiment 1 included 30 undergraduate and graduate students (age range: 18–24 years, 15 females) recruited from the participant pool at Hokkaido University who participated for pay or in exchange for course credit regardless of whether they had studied psychology. The present study included a larger number of participants than Bar and Neta (2006) and determined sample size based on a priori power analysis with G*Power 3.1 (Faul et al., 2007, 2009). The effect size was set as 0.62, which was approximated from Bar and Neta (2006), and the power was set as 0.95. All participants had normal or corrected-to-normal visual acuity. All experiments in the present study were approved by the Human Research Ethics Committee of Hokkaido University, Japan, and all participants provided written informed consent prior to participation in the experiment. Participants were not informed regarding the purpose of the study.

#### Stimuli and Apparatus

All stimuli were displayed on an LCD monitor (100 Hz refresh rate, 1,920 × 1,080 pixels, XL2411T; BenQ) that was controlled by MATLAB software using the Psychophysics Toolbox (Brainard, 1997; Kleiner et al., 2007; Pelli, 1997). Each stimulus was presented on a white square area (8.3° × 8.3°) on a black background at a viewing distance of 57 cm.

A total of 320 grayscale images (7.1° × 7.1°) of diverse real and abstract (i.e., meaningless) objects that were chosen from a part of the original set used by Bar and Neta (2006, 2007; available at https://faculty.biu.ac.il/∼barlab/stimuli.html) were used in Experiment 1. The stimuli comprise 70 pairs of real objects and 70 pairs of meaningless objects such that each pair consisted of two versions of items (curved vs. sharp-angled) with the same semantic content (e.g., curved chair/sharp-angled chair or curved pattern/sharpened pattern) that were counterbalanced across participants. Thus, each pair consisted of two images of real and meaningless objects that were equalized in the visual dimension except for contour, as described in Bar and Neta (2006). It was ensured that each participant viewed a variety of contour types across all pairs. In addition, 40 other real objects comprising roughly equal mixture of curved and sharp-angled features were included in the stimuli set. These 40 objects were used as control stimuli for the possible effects of semantic content or familiarity in the participants’ preferences. This procedure yielded the identical experimental design as Bar and Neta (2006) because the design for the real objects included the factor of contour with three levels (curved, sharp-angled, and control). For the meaningless objects, the experimental design included the factor of contour with two levels (curved or sharp-angled) because no control condition existed for meaningless objects. All pairs and control images were pooled in the same stimulus set, and the presentation order was randomized.

Real object images from the Bar and Neta online image set were selected as test stimuli, regardless of their semantic content. We included the odd-numbered files in the study. Ultimately, we presented images of a chair, a kettle, a tree, a brash, a clock, a cell phone, a bag, a glass, and a pot, among other objects. A flute, a spanner, an iron, pliers, a cherry, a pineapple, and a seal (all from the Bar and Neta set) were included as control images. The pilot study participants were familiar with all of the items such that they could name them. Cultural relevance was not considered when choosing the items.

#### Procedure

**Figure 1.**
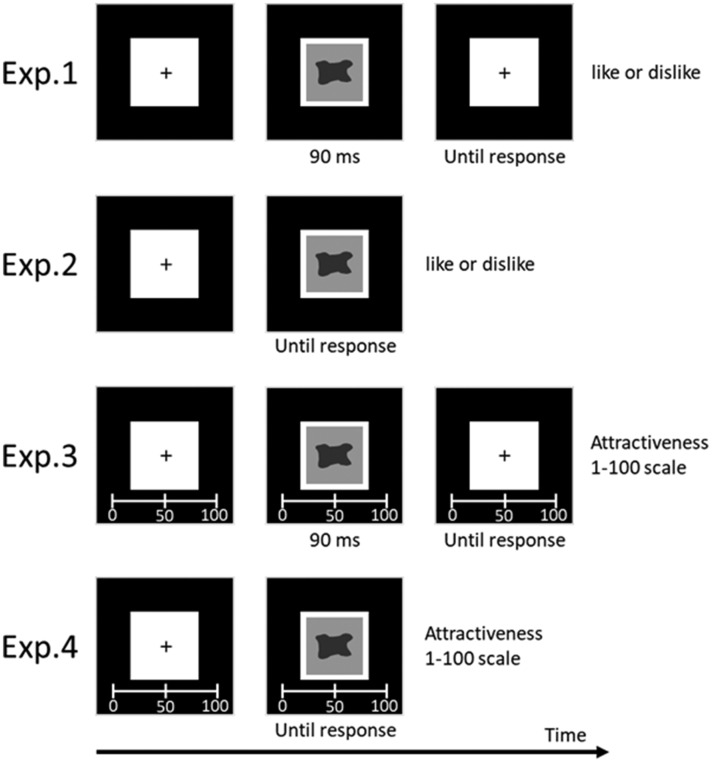
The Stimulus Sequence Used. One real and one abstract image were displayed in random order. Image examples can be found on Bar’s Cognitive Neuroscience Laboratory website.

All experimental procedures were conducted in a well-lit laboratory room. At the beginning of each trial, a black fixation cross (0.3° × 0.3°) was displayed on the white square area for 500 ms (Figure 1). Following the presentation of the fixation cross, each participant viewed one item from the set of stimuli that was either a curved, sharp-angled, or control object. Each stimulus was presented for 90 ms, and the order of presentation was randomized. Following the presentation of each stimulus, each participant evaluated their preference (*liking*) for the object using a like/dislike response by pressing the i/e key, respectively.

### Results and Discussion

**Figure 2.**
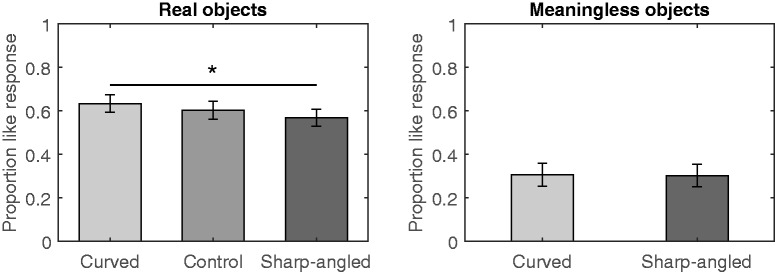
Mean Proportions of *Like* Responses in Experiment 1. This experiment used a like/dislike rating and a 90 ms presentation time. Error bars represent SEMs (*p < .05).

The data of one participant were excluded from the analyses because that individual fell within the 26- to 307-s long response time interval on more than 10 trials. All response times that deviated more than two standard deviations from the mean (0.03% of all trials) were excluded from further analyses because several trials were marked as having a long response time (i.e., more than 33 s). The proportion of *like* responses per participant was calculated separately for the contour type of real objects (curved, sharp-angled, or control) and for the meaningless object (curved or sharp-angled). The proportions of *like* responses for the real and meaningless objects are displayed in Figure 2. The means of the proportions for real objects were assessed with a one-way repeated measure analysis of variance (ANOVA) that included contour (curved, sharp-angled, or control) as the within-subject factor and revealed that contour type significantly affected a preference for real objects, *F*(2, 56) = 3.90, *p* = .026, ${\text{η}}_{\text{G}}^{\text{2}}$ = .02. Multiple comparisons using Holm’s method revealed that the participants preferred curved real objects over their sharp-angled counterparts, *t*(28) = 3.17, *p* = .011, *d* = 0.30. However, a paired *t* test revealed that the curvature effect was not significant for meaningless objects, *t*(28) = 0.13, *p* = .895, *d* = 0.01.

Experiment 1 aimed to determine whether participants would prefer curved to sharp-angled objects using a procedure that was identical to that used by Bar and Neta (2006), which observed a positive bias for curved objects regardless of whether the objects were real or meaningless drawings. The present findings partially replicated the curvature effect in that the effect occurred only for real objects. The present study also revealed that the curvature effect was reduced or eliminated when participants indicated likes or dislikes for meaningless objects. Moreover, the effect size of a preference for curved real objects was smaller (*d* = 0.24) than that reported by Bar and Neta (2006; *d* = 0.94), even though the percentages of like values between the studies were comparable (approximately 50–70%). Consistent with Bar and Neta (2006), the present study found that a preference for real objects was generally higher than that for meaningless objects, which indicates that the absence of semantic content decreased a preference for an object regardless of contour.

In Experiment 1, the participants viewed the objects during a brief presentation period of 90 ms. The present study hypothesized that the curvature effect for real objects would be reduced and that the effect for meaningless objects would be increased when the presentation times were increased, as previously reported (Corradi et al., 2018; Munar et al., 2015). Thus, in Experiment 2, the stimulus presentation time was extended to until-response (i.e., until the participant responded) using the same task as Experiment 1.

## Experiment 2

Experiment 2 was performed in exactly the same manner as Experiment 1 except that the presentation time of the object was until-response. In each trial, the participants viewed an image until they made a response and then immediately rated their preference using a forced-choice paradigm (like/dislike). A similar procedure was employed by Corradi et al. (2019b), who examined the effects of presentation time on the curvature effect. However, these authors employed a forced-choice response task that compared two objects simultaneously displayed on screen. In the present study, a critical change was made in that a single image was displayed on the screen to avoid the influence of difficulty when determining one’s preference (i.e., liking) for two similar stimuli that were displayed simultaneously (e.g., Kramer & Ritchie, 2016; Nachmias, 2011). Thus, the present experiment maintained the same procedures for response acquisition as those used by Bar and Neta (2006) in the original study.

### Method

Experiment 2 included 30 undergraduate and graduate students (age range: 18–23 years, 11 females) who all had normal or corrected-to-normal visual acuity. The stimuli, apparatus, and procedure used in this experiment were identical to those used in Experiment 1 expect that the exposure duration of the objects was until-response. The images were presented in the white square area (8.3° × 8.3°) on a black background in the center of the monitor at a viewing distance of 57 cm. Each participant viewed a variety of contour types across all pairs, which consisted of two versions of items (curved vs. sharp-angled) with the same semantic content. They also viewed all control objects.

### Results and Discussion

**Figure 3.**
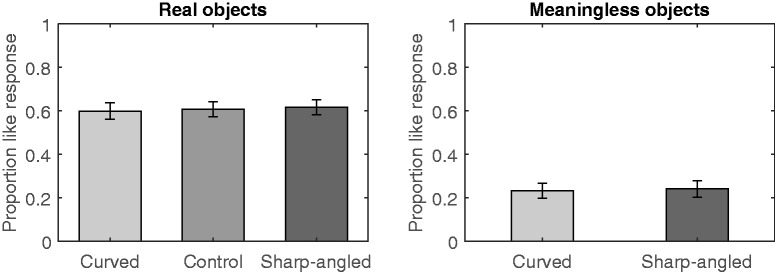
Mean Proportions of *Like* Responses in Experiment 2. This experiment used a like/dislike rating and an until-response. Error bars represent SEMs.

The proportion of *like* responses per participant was calculated separately for the contour type of real objects (curved, sharp-angled, or control) and for the meaningless objects (curved or sharp-angled). The means for the real and meaningless objects are displayed in Figure 3. The means of the proportions for real objects were assessed with a one-way repeated measure ANOVA that included contour (curved, sharp-angled, or control) as the within-subject factor and revealed that there was no significant curvature effect for real objects, *F*(2, 58) = 0.25, *p* = .781, η_G_^2^ = .002. Moreover, a paired *t* test revealed that the curvature effect was not significant for meaningless objects, *t*(29) = –0.27, *p* = .793, *d* = 0.04.

Experiment 2 revealed that the curvature effect for real objects was eliminated when the stimuli remained visible until the participant made a response. The present results contrasted sharply with the results of Experiment 1 in which the stimuli were only briefly exposed. Thus, the present study demonstrated that the human preference for real objects decreased under the until-response condition, which supports previous findings showing that an until-response presentation time decreases the preference for real curved objects (Corradi et al., 2018). In contrast, the preference for meaningless objects did not increase under the until-response condition, which is inconsistent with previous results showing an increased preference for meaningless objects when the presentation time is until-response (Corradi et al., 2018). The present findings suggest that the effects of presentation time fluctuate across studies and are not stable, as previously reported (e.g., Bar & Neta, 2006; Palumbo & Bertamini, 2016). However, similar to the results from Experiment 1, the present experiment was successful in replicating the findings of Bar and Neta (2006) in terms of showing that the preference for real objects was higher than for meaningless objects.

Both Experiments 1 and 2 used a like/dislike rating task to report preference for curved contours, as in previous studies (Bar & Neta, 2006; Corradi et al., 2018; Leder et al., 2011). However, recent studies (e.g., Bertamini et al., 2016; Palumbo & Bertamini, 2016) have employed different response measures, such as visual analog scales, to examine the curvature effect. Because response measures as well as presentation time may contribute to the modulation of human preference for objects, the use of different tasks with various measures to record responses might provide different results regarding the curvature effect. Thus, to examine the robustness of the curvature effect, the forced-choice response measure (like/dislike) was changed to a visual analog scale ranging from 1 to 100 in Experiments 3 and 4.

## Experiment 3

To examine the robustness of the curvature effect, Experiment 3 was designed to replicate Experiment 1 in terms of the 90-ms exposure duration, whereas the response measure was a visual analog scale that ranged from 1 to 100. Palumbo and Bertamini (2016) found that the robustness of the curvature effect remains when using two different response measures, that is, a two-alternative forced choice and a rating scale. However, it is possible that the human preference for curved objects could be modulated depending on the type of rating scale used to indicate preference. In fact, the present results from Experiment 2 differed from those reported by Corradi et al. (2019b), who used the same response measures as those used in the present study. However, because Palumbo and Bertamini (2016) did not include any real objects with an until-response presentation duration in their procedure, it is not possible to compare their results about the curvature effect directly with those of Bar and Neta (2006). Thus, in the present study, the participants in Experiment 3 performed the same task as in Experiment 1 but used an explicit scale that ranged from 1 to 100 (Bertamini et al., 2016; Palumbo & Bertamini, 2016) to rate their preferences, which would theoretically modulate the curvature effect.

### Method

Experiment 3 included 30 undergraduate and graduate students (age range: 18–24 years, 14 females) who all had normal or corrected-to-normal visual acuity. The stimuli, apparatus, and procedure used in this experiment were identical to those used in Experiment 1 except that the participants rated their preferences (i.e., attractiveness) for an object by clicking a specific point on a visual analog scale ranging from 1 to 100.

### Results and Discussion

**Figure 4.**
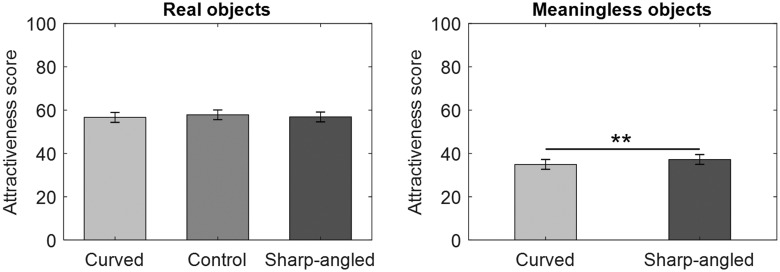
Mean *Attractiveness* Scores on a 1 to 100 Rating Scale in Experiment 3. This experiment used a 1 to 100 rating and a 90 ms presentation time. Error bars represent SEMs (**p < .01).

The rated scores were averaged for each contour type (curved, sharp-angled, or control) and item type (real or meaningless object) per participant. The group means are displayed in Figure 4. The means of the scores for real objects were assessed with a one-way repeated measure ANOVA that included contour (curved, sharp-angled, or control) as a within-subject factor and revealed no significant curvature effect, *F*(2, 58) = 1.41, *p* = .252, ${\text{η}}_{\text{G}}^{\text{2}}$ = .002. A paired *t* test revealed that the main effect of contours was significant for meaningless objects, *t*(29) = –2.89, *p* = .007, *d* = 0.19. In contrast to previous findings (Bar & Neta, 2006), the preference for sharp-angled objects without semantic content was higher than that for curved objects.

Experiment 3 examined whether participants would prefer objects with curved contours to those with sharp-angled contours following a 90-ms exposure duration when using a visual analog scale as a response measure. This measure was identical to those used in previous studies (Bertamini et al., 2016; Palumbo & Bertamini, 2016) showing that humans prefer curved contours when using this type of scale. However, consistent with the results of Experiment 2 in the present study, there was no reliable curvature effect for real objects. Furthermore, Experiment 3 revealed a reverse pattern of results such that the preference for sharp-angled meaningless objects exceeded that of curved ones. These findings are inconsistent with those of previous studies showing that the human preference for curved contours is a robust effect (e.g., Corradi et al., 2018), even when using different tasks (Palumbo & Bertamini, 2016). Thus, the present study did not replicate the findings of Bar and Neta (2006) regarding the curvature effect when using a different response measure than that used in the original study. Similar to the results of Experiments 1 and 2, the results of Experiment 3 support the notion that the preference for real objects is higher than that for meaningless things (Bar & Neta, 2006, 2007; Leder et al., 2011).

## Experiment 4

To examine further the robustness of the curvature effect, Experiment 4 was designed to replicate Experiment 2 in terms of the until-response condition but used a visual analog scale that ranged from 1 to 100 as the response measure. The until-response condition was used in Experiment 4 because it was not a major focus of a previous relevant study (Palumbo & Bertamini, 2016) that compared different tasks (two-alternative forced-choice task vs. rating scale task) as their primary interest.

### Method

Experiment 4 included 30 undergraduate and graduate students (age range: 18–38, 10 females) who all had normal or corrected-to-normal visual acuity. The stimuli, apparatus, and procedure used in this experiment were identical to those used in Experiment 2 except that the participants rated their preference (i.e., attractiveness) for an object by clicking a specific point on a visual analog scale ranging from 1 to 100.

### Results and Discussion

**Figure 5.**
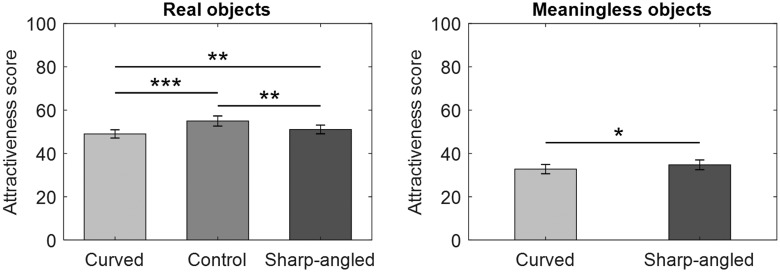
Mean *Attractiveness* Scores on a 1 to 100 Rating Scale in Experiment 4. This experiment used a 1 to 100 rating and an until-response. Error bars represent SEMs (*p < .05, **p < .01, ***p < .001).

The rated scores were averaged for each contour type (curved, sharp-angled, or control) and item type (real object or meaningless object) per participant. The group means are displayed in Figure 5. The means of the scores for real objects were assessed with a one-way repeated measure ANOVA that included contour (curved, sharp-angled, or control) as the within-subject factor and revealed a significant effect of contour, *F*(2, 58) = 15.68, *p* < .001, ${\text{η}}_{\text{G}}^{\text{2}}$ = .05. Multiple comparisons using Holm’s method revealed that the participants preferred the control objects over real curved objects, *t*(29) = 4.71, *p* < .001, *d* = 0.51, control objects over real sharp-angled objects, *t*(29) = 3.25, *p* = .003, *d* = 0.33, and real sharp-angled objects over their curved counterparts, *t*(29) = 3.02, *p* = .005, *d* = 0.19. A paired *t* test revealed that the effect of contours was significant for meaningless objects, *t*(29) = –2.50, *p* = .018, *d* = 0.17. The preference for sharp-angled objects without semantic content increased compared with that for curved objects.

Consistent with the results of Experiment 1, there was no reliable curvature effect for real objects. However, similar to the results of Experiment 3, there was a reverse pattern of results such that the preference for sharp-angled meaningless objects and real objects exceeded that for curved ones. In addition, the preference for real objects was higher than that for meaningless objects, which is consistent with the results of Experiments 1 to 3.

## General Discussion

The primary aim of the present study was to examine the robustness of the curvature effect by examining various factors, such as semantic content, response measures, and presentation time, which can influence the human preference for an object. The present results were not consistent with those of previous studies investigating the curvature effect (Bar & Neta, 2006; Corradi et al., 2018; Palumbo & Bertamini, 2016) in that the present participants preferred curved items only under limited conditions. Specifically, when participants had to choose a preference using a like/dislike response, the proportion of likes increased only when real objects were presented briefly (i.e., 90 ms). However, the effect size in the present study was substantially smaller than those reported by Bar and Neta (2006). In addition, contrary to the results reported by Palumbo and Bertamini (2016), the present results suggest that the curvature effect was modulated by the type of measure used to assess the task, that is, a like/dislike response versus a visual analog scale ranging from 1 to 100. Specifically, when the participants indicated preferences using like/dislike ratings under the until-response condition (Experiment 2), using a rating scale following brief exposure (Experiment 3), and using a rating scale under the until-response condition (Experiment 4), their preferences were not biased toward the curved objects. In fact, reversed patterns of results were observed in Experiments 3 and 4 such that preference was biased toward the sharp-angled objects. Taken together, these findings suggest that the preference for curved objects was situation-dependent and that some of these effects were modulated by semantic content, response measure, and presentation time.

Previous findings regarding the robustness of the curvature effect provide strong evidence for the use of common visual features across a broad range of visual stimuli, age, sex, and/or species (Corradi et al., 2019; Gómez-Puerto et al., 2016; Jadva et al., 2010). As a result, the application of the curvature effect could potentially impact product design and packaging to facilitate commercial success. However, the present results did not support a generalization of the effects of curved contours to Japanese observers. Thus, the discrepancies between the present results and previous studies (Bar & Neta, 2006; Corradi et al., 2018; Palumbo & Bertamini, 2016) regarding the robustness of the curvature effect remain a matter of debate.

The present results validated preferences based on semantic content, response measure, and presentation time. First, the preference for real objects was higher than that for meaningless objects regardless of presentation time or response measure, and, thus, the difference between these two classes of objects must be related to semantic content. Taken together, the present findings support the notion that semantic content impacts one’s preference for an object (Leder et al., 2011). Second, using a 1 to 100 rating scale reduced the preference for curved objects regardless of semantic content, which is inconsistent with previous findings showing that the curvature effect is stable across different response measures (Bertamini et al., 2016; Palumbo & Bertamini, 2016). The effects of the rating scale might be related to the notion that this type of measure facilitates explicit ratings based on object meaning (Bertamini et al., 2016; Dewey & Knoblich, 2014), and, thus, subjects might underestimate information extracted based on low-level processing (see Bar and Neta, 2006, 2007). However, the present study found that the preference for curved meaningless objects did not increase when using the rating scale, which indicates that semantic content was not a critical factor for preference when using the rating scale. Third, consistent with previous findings (Corradi et al., 2018; Munar et al., 2015), the long presentation time used in the present study decreased the curvature effect for real objects. However, the increased preference for curved meaningless objects following a long exposure (Corradi et al., 2018) was not supported by the present findings. Thus, these factors can partially explain the low preference for curved objects.

A recent study on the curvature effect (e.g., Gómez-Puerto et al., 2016) acknowledged the lack of studies investigating how cultural differences might influence this phenomenon. Thus, cultural differences should be considered as a possible modulator of the curvature effect. In fact, the curvature effect has been primarily studied using Western populations (Corradi et al., 2019a), except for a few studies that used non-Western populations, such as in Ghana (e.g., Gómez-Puerto et al., 2018). Studies (Cho et al., 2018; Tzeng et al., 1990) have addressed cultural differences between Asians (including Japanese) and other populations, but the topics thereof were not directly relevant to the curvature effect. The present study demonstrated a preference for curved objects, albeit weak, in Asian (i.e., Japanese) observers. This finding is consistent with that of Zhang et al. (2006), who showed that Japanese populations perceived curved shapes to be more attractive than did other populations. The important finding of the present study is that the effect could only be replicated when using the original task of Bar and Neta (2006). It is possible that the discrepancies between the present and previous findings were due to the tendency for Japanese observers to examine objects holistically rather than analytically, and, thus, the sharp-angled edges may not have contributed to the perception of a threat. In a related study, Okamura and Ura (2018) found that drawings of squares but not circles are associated with competence. Therefore, cultural perspectives should be taken into account when interpreting differences in replications of the curvature effect.

Interestingly, in the present study, inverse trends showing a preference against the curvature effect were observed when the participants used a rating scale as a response measure (Experiments 3 and 4). More specifically, the present participants preferred sharp-angled objects over curved objects, and this preference was consistent across the types of objects, except for after a shorter (90 ms) exposure duration. Experiments 3 and 4 had effect sizes (favoring sharp-angled objects) of 0.17 and 0.19, respectively, which were smaller than the effect size of Experiment 1 (*d* = 0.30; favoring curved objects). Thus, the inverse effects seemed to be smaller than the curvature trends. Nevertheless, these results indicate a stable preference for sharp-angled objects compared with curved objects, which is inconsistent with the findings of Palumbo and Bertamini (2016) who used a very similar experimental methodology to compare different tasks. In fact, Corradi et al. (2019a), who demonstrated the robustness of the curvature effect in Western adult subjects, suggested that a preference of individuals substantially differs across people. Specifically, that study included populations who preferred real and abstract images with sharp-angled contours over curved contours.

It should be noted that the terms indicating *preferences* were not identical among experiments such that we cannot rule out an effect of semantic meaning on the results. For example, in Experiments 1 and 2, objects were rated in terms of how much they were *liked* while in Experiments 3 and 4, participants rated items in terms of *attractiveness*. It could be useful to explore whether the inverse trends with respect to the curvature effect stemmed from differences in the terms employed in our instructions. In contrast, Munar et al. (2015) avoided words such as *liking* or *preferring* such that ratings were not affected by differences in semantic meaning.

The abstract stimuli employed in this study may have exerted other unwanted effect. The stimuli were not controlled, in that their configuration (e.g., distance to the top and bottom of the frame, outer frame shape, and color contrast) varied. In terms of contrast, Palumbo and Bertamini (2016), Corradi et al. (2019b), and Cotter et al. (2017) used abstract stimuli in which only the contour line varied (curved vs. sharp-angled). Thus, differences in configuration may have affected participants’ preference in the present study.

In summary, the present study found that the curvature effect depended on the semantic content of the object, the measure used to record the response to the object, and the presentation time of the object in a non-Western Japanese population. The present study replicated the curvature effect using the procedure of Bar and Neta (2006). However, when participants viewed real objects briefly (90 ms), the robustness of the curvature effect was not observed across different tasks. These findings suggest that contour is not the dominant characteristic contributing to a preference for objects, at least in Japanese observers. The semantic meanings of objects and/or other visual features such as symmetry, brightness, typicality, and perceptual fluency (Lakens et al., 2013; Leder et al., 2011; Perrett, 2012; Perrett et al., 1999; Winkielman et al., 2006) might underlie a preference for, as well as the influence of, contours.
